# KLF5-induced BBOX1-AS1 contributes to cell malignant phenotypes in non-small cell lung cancer via sponging miR-27a-5p to up-regulate MELK and activate FAK signaling pathway

**DOI:** 10.1186/s13046-021-01943-5

**Published:** 2021-04-30

**Authors:** Jiang Shi, Chao Yang, Jinlu An, Dexun Hao, Cong Liu, Jumin Liu, Jing Sun, Junguang Jiang

**Affiliations:** 1 Department of Geriatric Respiratory Medicine, the First Affiliated Hospital of Zhengzhou University, Zhengzhou, 450052 China; 2 Department of Neurology, the First Affiliated Hospital of Zhengzhou University, Zhengzhou, 450052 China

**Keywords:** Non-small cell lung cancer, lncRNAs, BBOX1-AS1, miR-27a-5p, MELK, KLF5

## Abstract

**Background:**

Non-small cell lung cancer (NSCLC) is a major histological subtype of lung cancer with high mortality and morbidity. A substantial amount of evidence demonstrates long non-coding RNAs (lncRNA) as critical regulators in tumorigeneis and malignant progression of human cancers. The oncogenic role of BBOX1 anti-sense RNA 1 (BBOX1-AS1) has been reported in several tumors. As yet, the potential functions and mechanisms of BBOX1-AS1 in NSCLC are obscure.

**Methods:**

The gene and protein expression was detected by qRT-PCR and western blot. Cell function was determined by CCK-8, colony forming, would healing and transwell assays. Bioinformatics tools, ChIP assays, dual luciferase reporters system and RNA pull-down experiments were used to examine the interaction between molecules. Subcutaneous tumor models in nude mice were established to investigate in vivo NSCLC cell behavior.

**Results:**

BBOX1-AS1 was highly expressed in NSCLC tissues and cells. High BBOX1-AS1 expression was associated with worse clinical parameters and poor prognosis. BBOX1-AS1 up-regulation was induced by transcription factor KLF5. BBOX1-AS1 deficiency resulted in an inhibition of cell proliferation, migration, invasion and EMT in vitro. Also, knockdown of BBOX1-AS1 suppressed NSCLC xenograft tumor growth in mice in vivo. Mechanistically, BBOX1-AS1 acted act as a competetive “sponge” of miR-27a-5p to promote maternal embryonic leucine zipper kinase (MELK) expression and activate FAK signaling. miR-27a-5p was confirmed as a tumor suppressor in NSCLC. Moreover, BBOX1-AS1-induced increase of cell proliferation, migration, invasion and EMT was greatly reversed due to the overexpression of miR-27a-5p. In addition, the suppressive effect of NSCLC progression owing to BBOX1-AS1 depletion was abated by the up-regulation of MELK. Consistently, BBOX1-AS1-mediated carcinogenicity was attenuated in NSCLC after treatment with a specific MELK inhibitor OTSSP167.

**Conclusions:**

KLF5-induced BBOX1-AS1 exerts tumor-promotive roles in NSCLC via sponging miR-27a-5p to activate MELK/FAK signaling, providing the possibility of employing BBOX1-AS1 as a therapeutic target for NSCLC patients.

**Supplementary Information:**

The online version contains supplementary material available at 10.1186/s13046-021-01943-5.

## Background

According to the statistic data from International Agency for Research on Cancer, lung cancer still ranks first in cancer morbidity and mortality all over the world, accounting for approximately 11.6% of the total cases and 18.4% of the total cancer deaths, respectively [1]. Nearly 85% of lung cancer patients are diagnosed as non-small cell lung cancer (NSCLC), of which lung adenocarcinoma (LUAD) and lung squamous cell carcinoma (LUSC) are the most common histological subtypes [2]. In addition to cigarette smoking, occupational carcinogens and air pollution are also regarded to be associated with this fatal disease [3]. Due to the deficiency of early detection biomarkers and obvious clinical symptoms, most patients are diagnosed at an advanced-stage [4]. A report on cancer survival during the most recent time period (2007–2013) shows an unfavorable prognosis for lung cancer patients, with a 5-year survival rate of merely 5% in distant stage [5]. Although recent advances in targeted therapy and immunotherapy have brought an exciting possibility of long term responses in advanced NSCLC [6, 7], elucidating the deep-going pathological mechanisms behind NSCLC progression is necessary to develop novel molecular targets and prolong patient survival.

Long non-coding RNAs (LncRNAs), non-protein-coding transcripts with a length greater than 200 nucleotides, regulate the expression of neighboring or distal genes ranging from integrating chromatin remodeling complexes to tuning transcription and post-transcriptional processes [8]. LncRNAs participate in carcinogenesis and metastasis in a variety of human malignancies through serving as signaling transducers, enhancers, structure scaffolds or molecular decoys by physically interacting with other RNA species, proteins or chromatin [9, 10]. Due to high specificity and easy detection in the tissues, serum and plasma, lncRNAs have been potentially explored as diagnostic and prognostic biomarkers and therapeutic targets for cancer patients [11]. A growing list of lncRNAs are reported as tumor suppressors or drivers in NSCLC via impacting fundamental cellular processes, such as proliferation, survival, apoptosis, migration, invasion and epithelial to mesenchymal transition (EMT) [12, 13]. For instance, lncRNA LINC01234 conferred an aggressive phenotype to NSCLC by sponging miR-340-5p/miR-27b-3p in the cytoplasm and inhibiting BTG2 expression in the nucleus [14]. Hypoxia-induced lncRNA AC020978 facilitates cell proliferation, migration and glycolytic metabolism in NSCLC through controlling PKM2/HIF-1α axis [15]. BBOX1 anti-sense RNA 1 (BBOX1-AS1) was recently clarified as an oncogenic lncRNA in several human tumors, including gastric cancer [16], cervical cancer [17], ovarian cancer [18] and colorectal cancer [19]. Through mining the microarray data of GEO database including GSE18842 and GSE19188, BBOX1-AS1 expression was found to be higher in NSCLC tumor tissues than that in adjacent non-cancerous tissues. Its detailed functions and underlying mechanisms, however, remain undefined.

It is now well understood that some lncRNAs could act as competing endogenous RNAs (ceRNAs) by binding to miRNA to reduce their inhibitory effects on target mRNAs [20, 21]. Evidence to support this concept is also found in lung tumors [22, 23]. Here, a series of clinical and functional experiments were conducted to uncover the biological significance of BBOX1-AS1 in NSCLC, as well as whether the involvement of BBOX1-AS1 in NSCLC was attributed to the similar ceRNA regulating cascades.

In this study, we demonstrated that KLF5-induced BBOX1-AS1 could serve as a molecular sponge for miR-27a-5p to up-regulate MELK expression and activate FAK signaling, thereby facilitating NSCLC cell proliferation and metastasis in vitro and in vivo. Our data highlights the potential clinical value of BBOX1-AS1 as prognostic biomarker and therapeutic target in NSCLC.

## Materials and methods

### Tissue samples

Tumor tissues and adjacent non-cancerous tissues were surgically obtained from 76 patients with a histopathological diagnosis of NSCLC at the First Affiliated Hospital of Zhengzhou University between June 2014 and May 2017. None of patients received preoperative anticancer treatment. The acquired tissues specimens were quickly frozen in liquid nitrogen and then conserved in a refrigerator at − 80 °C. The experimental procedures involving human subjects were conducted according to the principles of Declaration of Helsinki and approved by the Institutional Ethics Committee of the First Affiliated Hospital of Zhengzhou University. Each patient signed the informed written consent prior to participating in this research.

### Cell culture

An immortalized human bronchial epithelial cell line BEAS-2B and NSCLC cells (A549, H1975, SK-MES-1 and H520) were obtained from the Institute of Biochemistry and Cell Biology of the Chinese Academy of Sciences (Shanghai, China). NSCLC cells were grown in RPMI 1640 medium (Thermo Fisher Scientific, Waltham, MA, USA) with 10% fetal bovine serum, 100 U/mL penicillin and 100 μg/mL streptomycin. BEAS-2B cells were maintained in Bronchial Epithelial Basal Medium (Lonza, Walkersville, Maryland, USA). All cells were cultured in a 37 °C incubator with humidified atmosphere of 5% CO_2_. Cells were negative for mycoplasma contamination and routinely subcultured at 80% confluence.

### Cell transfection

Small interfering RNAs (siRNAs) specifically targeting BBOX1-AS1 (si-BBOX1-AS1#1, si-BBOX1-AS1 #2) or MELK (si-MELK) and corresponding non-specific siRNA (si-NC) were obtained from GenePharma (Shanghai, China). MiR-27a-5p mimic (miR-27a-5p), miR-27a-5p inhibitor (inh-miR-27a-5p) and their corresponding negative nonsense sequences (miR-NC/inh-NC) were purchased from RiboBio (Guangzhou, China). BBOX1-AS1-overexpressing plasmid (BBOX1-AS1), MELK-overexpression vector (MELK) and negative control pcDNA3.1 (Vector) were provided by Geneseed (Guangzhou, China). When cells grew to about 70–80% confluence, transfection was performed using Lipofectamine 3000 reagent (Invitrogen, Carlsbad, CA, USA) according to the manufacturer’s guidance. At 48 h after transfection, cells were harvested for further analysis.

### RNA isolation and quantitative real-time PCR (qRT-PCR)

Total RNA extraction from tissue specimens and cells were conducted by using TRIzol Reagent (Invitrogen) following the supplier’s instructions. For cDNAs synthesis of BBOX1-AS1 or MELK, 1 μg of total RNA was subjected to reverse transcription by using RevertAid First Strand cDNA Synthesis Kit (Thermo Fisher Scientific, Waltham, MA, USA). For miR-27a-5p, cDNAs were generated from total RNA with TaqMan MicroRNA reverse transcription kit (Applied Biosystems, Foster City, CA, USA). Quantitative PCR amplification was performed with LightCycler 480 SYBR Green I Master mix (Roche, Mannheim, Germany) on a LightCycler 480 Instrument II (Roche). The 2^–ΔΔCT^ method was applied to evaluate the relative gene expression. Glyceraldehyde-3-phosphate dehydrogenase (GAPDH) was used as an endogenous control to normalize against BBOX1-AS1 or MELK, while U6 snRNA served as an internal reference for miR-27a-3p. The primer sequences were listed in Additional file 1: Table S1.

### Western blot assay

NSCLC cells were lysed with RIPA lysis buffer (Beyotime, Shanghai, China) containing a protease inhibitor cocktail (Roche, Basel, Switzerland) to extract the total protein. Protein concentration was determined by DC protein assay kit (Bio-Rad, Hercules, CA, USA). Equivalent protein (25 μg) from each group was loaded on 10% SDS-PAGE gel, transferred onto 0.22 μm PVDF membranes and blocked in 5% skimmed milk to minimize non-specific binding of antibodies. Then, the membranes were separately probed with primary antibodies and further incubated with HRP-conjugated secondary for 2 h at room temperature. The protein blots were visualized by using an enhanced chemiluminescence detection kit (ECL Plus; Millipore, Billerica, MA, USA). Images were acquired with Image Quant Las 4000 system (GE Healthcare, Chicago, IL, USA) and band intensities were quantified by ImageQuant TL software (GE Healthcare). Antibodies against E-cadherin, N-cadherin, Vimentin and GAPDH were purchased from Cell Signaling Technology (Danvers, MA, USA). Antibodies against MELK, p-FAK and FAK were obtained from Abcam (Cambridge, MA, USA).

### CCK-8 assay

Cell proliferation potential was evaluated by using CCK-8 kit (Dojindo, Kumamoto, Japan). Brief, transfected NSCLC cells were seeded into 96-well plates at a density of 2.5 × 10^3^ cells/well and incubated under standard condition for 1, 2, 3 and 4 days. After adding 10 μl of CCK-8 solution, the plates were put back to the incubator for incubation of another 2.5 h. The absorbance at 450 nm was recorded by a microplate reader (Bio-Rad, Hercules, CA, USA) to depict the growth curves.

### Colony formation assay

Transfected NSCLC cells were inoculated in 6-well plates (800 cells per well) and cultured for another two weeks. During the culture processes, the medium was refreshed twice a week. At last, cells were washed with PBS, fixed with methanol and stained with crystal violet for 20 min. Images were photographed with a Leica DMIL inverted light microscope (Leica Microsystems, Germany) and visible colonies were then counted.

### Wound healing assay

Transfected NSCLC cells were inoculated in 6-well plates and grew to a 100% confluence. Then, the culture medium was discarded and a linear wound was scratched across the monolayer using a sterile 200 μl pipette tip. After removing the non-adherent cells and debris, the refresh medium was added for 48 h incubation. Images were taken by an inverted microscope at 0 h and 48 h after the wounds were generated. The percentage of wound closure (%) = (width on day 0 - with on day 48)/width on day 0 × 100%.

### Transwell invasion assay

Transwell chambers (Costar, Corning Inc., NY, USA) with 8 μm pore size membranes were applied to examine cell invasion ability. The membranes of the inserts were pre-coated with Matrigel matrix at 24 h prior to cell seeding. A total of 2 × 10^5^ NSCLC cells in 200 μL serum-free medium were added to the upper chamber, while the bottom chamber was filled with 600 μL complete serum-containing medium. After 24 h of incubation, the non-invasive cells were wiped off from the upper surface of the filter by using a cotton swab. At the same time, cells that had travelled through the membrane were fixed with methanol, stained with crystal violet and photographed by an optical microscope with digital camera. The average number of invasive cells was calculated from five randomly selected fields.

### Subcellular fractionation

PARIS kit protein and RNA isolation system (Life Technologies, Carlsbad, CA, USA) was applied to isolate and purify the nuclear and cytoplasmic RNA from NSCLC cells in accordance with the manufacturer’s guidance. qRT-PCR was used to measure the expression of BBOX1-AS1 from nuclear and cytoplasmic fractions, with U6 and GAPDH as respective reference.

### Bioinformatics analysis

Microarray data were downloaded from GEO datasets (GSE18842, GSE19188, GSE135918, GSE29250 and GSE33532). GEPIA (https://gepia.cancer-pku.cn/index.html) and starBase (https://starbase.sysu.edu.cn/) were used to analyze the gene expression level and gene correlation. Kaplan-Meier Plotter (https://kmplot.com/analysis/) was applied to assess the effect of genes on patient prognosis in lung cancer. JASPAR (https://jaspar.genereg.net/) was utilized to predict the binding sites between KLF5 and BBOX1-AS1 promoter. DIANA-LncBase (https://carolina.imis.athena-innovation.gr/diana_tools/web/index.php?r=lncbasev2%2Findex) and LncBook (https://bigd.big.ac.cn/lncbook/index) were used to predict the candidate miRNAs that contain BBOX1-AS1 binding sites. TargetScan (https://www.targetscan.org/vert_72/) and miRDB (https://mirdb.org/) were employed to predict the potential targets of miR-27a-5p. The subcellular localization of BBOX1-AS1 was predicted by using lncLocator (https://www.csbio.sjtu.edu.cn/bioinf/lncLocator).

### Chromatin immunoprecipitation (ChIP) assay

EZ-ChIP Chromatin immunoprecipitation kit (Millipore, Bedford, MA, USA) was used to perform ChIP assay. In brief, NSCLC cells were treated with 1% formaldehyde for 15 min to generate DNA-protein cross-linking. Subsequently, cells were lysed and sonicated to obtain chromatin fragments with an average size of 200 to 500 bp. Then, magnetic protein A beads coupled with KLF5 antibody (Abcam, Cambridge, MA, USA) were used to precipitate the chromatin fragments. Normal rabbit IgG was used as a negative control. Finally, the precipitated DNA was subjected to qRT-PCR assay.

### Dual-luciferase reporter assay

The wild type (wt) sequences of BBOX1-AS1 or MELK-3’UTR harboring miR-27a-5p binding sites were subcloned to the downstream of the Firefly luciferase coding region in pmirGLO vector (Promega, Madison, WI, USA), generating BBOX1-AS1-wt and MELK-wt reporter. The mutant version of BBOX1-AS1 or MELK-3’UTR (BBOX1-AS1-mut or MELK-mut) was created by using QuikChange XL Site-Directed Mutagenesis kit (Agilent Technologies, Santa Clara, CA, USA) to change the nucleotides complementary to miR-27a-5p. The constructed luciferase reporters were transfected into NSCLC cells together with miR-NC or miR-27a-5p. After 48 h, the luciferase activities were examined with a Dual-Luciferase reporter assay system (Promega). The relative luciferase activity was determined by normalizing the firefly luciferase activity against the Renilla luciferase activity.

### Biotin-coupled miRNA pull-down assay

The biotinylated miRNA mimic (Biotin-miR-27a-5p) and negative control (Biotin-NC) were purchased from GenePharma (Shanghai, China). NSCLC cells were transfected with biotinylated RNAs and cultured for 48 h at 37 °C. Then, cells were harvested and lysed in the lysis buffer, followed by incubation with streptavidin-coated magnetic beads at 4 °C for 2 h. The enrichment of BBOX1-AS1 or MELK mRNA in biotin-coupled RNA complex was detected by qRT-PCR.

### In vivo xenograft assays

All animal procedures strictly conformed to the National Institutes of Health guide for the care and use of Laboratory animals and authorized by the Institutional Animal Care and Use Committee of the First Affiliated Hospital of Zhengzhou University. Male BALB/c nude mice (6 weeks old, 20–24 g) were purchased from Shanghai SLAC Laboratory Animal Center (Shanghai, China) and allowed to acclimatize for at least one week prior to experiments. They were caged in a specific pathogen-free animal facility and fed with standard rodent food and water ad libitum. Mice were subcutaneously injected with A549 cells (6 × 10^6^) with lentivirus vectors carrying sh-BBOX1-AS1 or sh-NC at the left armpit. Tumor size was measured and recorded every 5 days. Tumor volume was calculated with the formula V = 0.5ab^2^, where ‘a’ and ‘b’ represent the long and the short diameters of the tumor, respectively. At 27 days post-inoculation, mice were killed and tumor masses were removed for subsequent assays.

### Immunohistochemistry (IHC)

Xenograft tumor tissues excised from mice were washed with PBS, fixed with 4% paraformaldehyde, embedded in paraffin and cut into 4-μm slices. Then, the slices were incubated with primary antibody against Ki-67 (Santa Cruz Biotechnology, Dallas, TX, USA) or PCNA (Santa Cruz Biotechnology) at 4 °C overnight, followed by treated with HRP-conjugated secondary antibody (Santa Cruz Biotechnology) for 1 h at room temperature. After stained with diaminobenzidine and haematoxylin, the tissue sections were visualized under a light microscope.

### Statistical analysis

Statistical evaluations were done using the GraphPad Prism 7 (GraphPad Software, Inc., La Jolla, CA, USA). All data were shown as mean ± standard deviation (SD) from at least 3 independent experiments. Gene expression difference between tumor tissue samples and adjacent normal tissues were determined using a paired *t* test. Student’s *t* test was used to compare the difference between two groups, while one-way analysis of variance (ANOVA) was applied for comparison among three groups or more. The correlation between BBOX1-AS1 expression and clinicopathological features was analyzed with χ2 test. The overall survival was estimated by using Kaplan-Meier method and compared with log-rank test. Statistically significant data were presented as *P < 0.05, **P < 0.01 and ***P < 0.001.

## Results

### BBOX1-AS1 is up-regulated in NSCLC and represents a poor prognosis

**Fig. 1:**
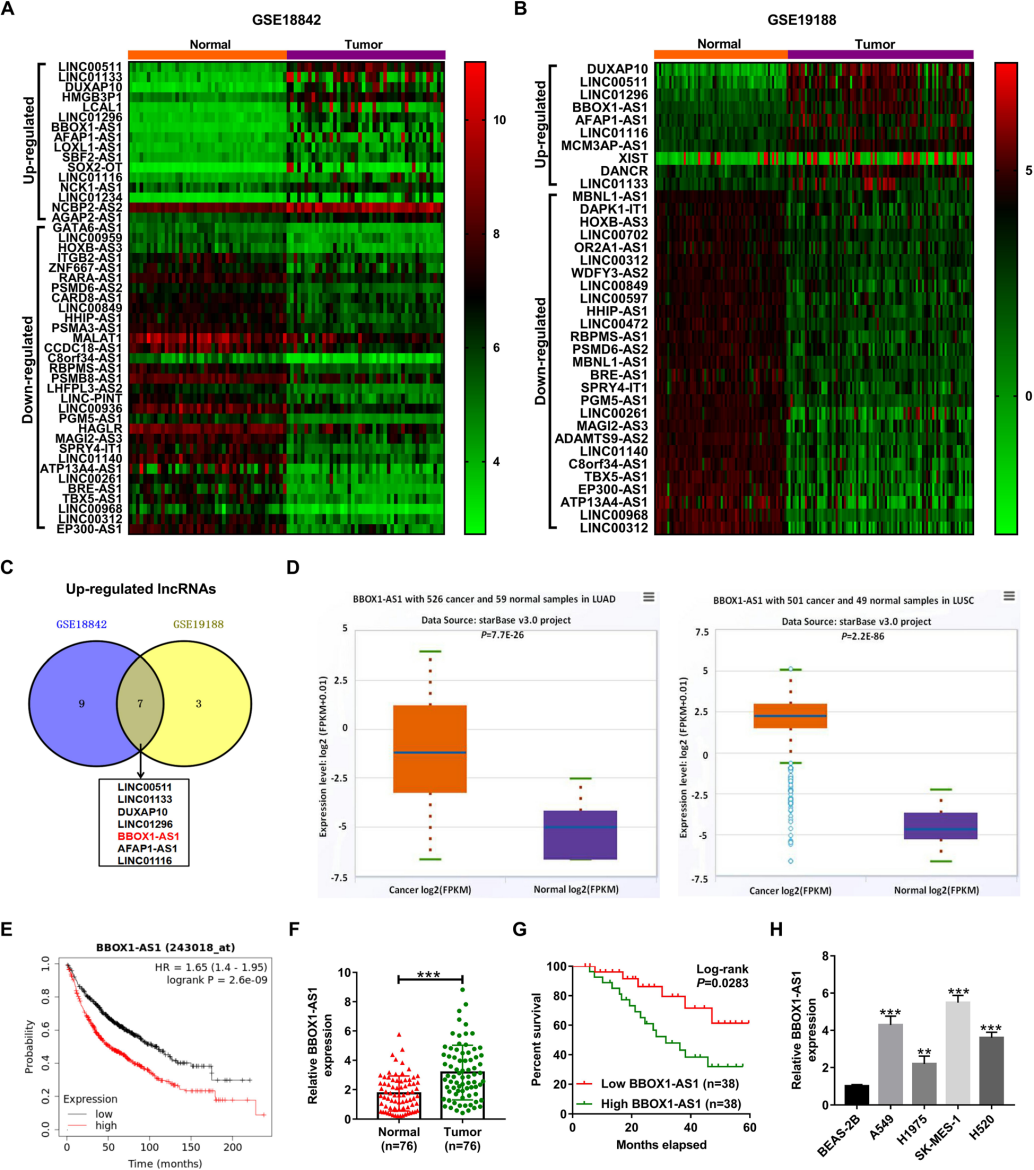
**BBOX1-AS1 is up-regulated and predicts an inferior prognosis in NSCLC. a** and **b** Heatmap of differentially expressed lncRNAs in lung cancer based on microarray datasets (GSE18842 and GSE19188). **c** Venn diagram displaying seven up-regulated lncRNA in NSCLC in both GSE18842 and GSE19188. **d** Expression of BBOX1-AS1 in LUAD and LUSC based on starBase Pan-Cancer Analysis Platform. **e** The correlation between BBOX1-AS1 expression and prognosis of NSCLC patients was evaculated by the Kaplan-Meier plotter database. **f** qRT-PCR analysis of BBOX1-AS1 expression in tumor tissues and corresponding non-cancerous tissues from a cohort of 76 ESCC patients. **g** Kaplan-Meier survival curve was used to examine the overall survival of NSCLC patients with high/low expression of BBOX1-AS1. **h** BBOX1-AS1 expression was measured by qRT-PCR in bronchial epithelial cell line BEAS-2B and four NSCLC cells. **P < 0.01, ***P < 0.001

In order to identify the differentially expressed lncRNAs in NSCLC, two sets of lung cancer microarray data (GSE18842 and GSE19188) were downloaded from GEO database. The aberrantly expressed lncRNAs were displayed in heatmap (Fig. 1a and b). According to the intersection of up-regulated lncRNAs in GSE18842 and GSE19188, seven lncRNAs (LINC00511, LINC01133, DUXAP10, LINC01296, BBOX1-AS1, AFAP1-AS1 and LINC01116) were found to be highly expressed in NSCLC tumor tissues in both datasets (Fig. 1 c). Among these lncRNAs, BBOX1-AS1 was selected for further analysis due to that its functions and mechanisms in NSCLC were still unclear. By using the starBase Pan-Cancer Analysis Platform, BBOX1-AS1 expression was observed to be increased in both LUAD and LUSC tissues (Fig. 1d). Consistently, bioinformatics tool GEPIA also presented a rise of BBOX1-AS1 expression in LUSC tissues (Additional file 1: Fig. S1A). As depicted by survival analysis platforms Kaplan-Meier Plotter, NSCLC patients with high BBOX1-AS1 expression had a worse prognosis compared to that with low BBOX1-AS1 expression (Fig. 1e). Subsequently, we performed qRT-PCR to determine the expression profile of BBOX1-AS1 in tumor tissues and adjacent non-cancerous tissues from 76 NSCLC patients. As a result, higher BBOX1-AS1 expression was observed in NSCLC tumor specimens than that in matched paraneoplastic tissues (Fig. 1f). Moreover, BBOX1-AS1 expression was positively associated with tumor size (*P* = 0.038), TNM stage (*P* = 0.006) and lymph node metastasis (*P* = 0.022) (Table 1). Kaplan-Meier survival curves demonstrated a lower survival for NSCLC patients expressing high level of BBOX1-AS1 (Fig. 1g). In addition, BBOX1-AS1 showed an increased expression in NSCLC cells in comparison with normal bronchial epithelial cell line BEAS-2B (Fig. 1h). Together, abnormally up-regulated BBOX1-AS1 might be associated with NSCLC progression and prognosis.

**Table 1:** Correlation between BBOX1-AS1 expression and clinicopathological parameters of NSCLC patients

Clinicopathological features	BBOX1-AS1 expression		*P* value
	Low (*n* = 38)	High (*n* = 38)	
Age			0.639
≤ 60	16	14	
> 60	22	24	
Gender			0.622
Male	11	13	
Female	27	25	
Tumor size			0.038*
< 5 cm	25	16	
≥ 5 cm	13	22	
TNM stage			0.006*
I/II	26	14	
III/IV	12	24	
Lymph node metastasis			0.022*
Negative	23	13	
Positive	15	25	
Differentiation grade			0.454
Well/moderate	28	25	
Poor	10	13	
Histology type			
Adenocarcinoma	20	18	0.646
Squamous carcinoma	18	20	

*P < 0.05

### Transcription factor KLF5 binds to BBOX1-AS1 promoter to activate its transcription

**Fig. 2:**
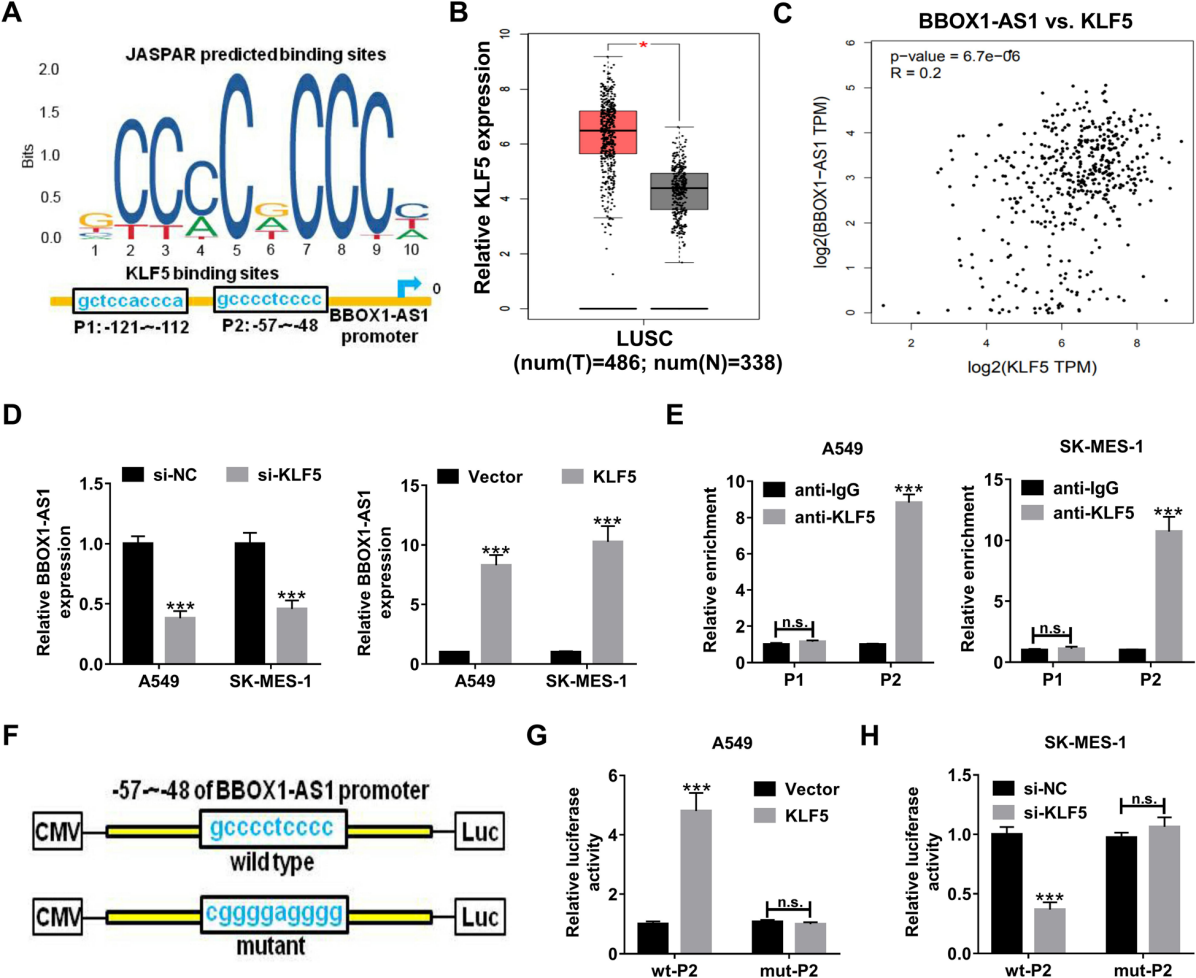
**KLF5 transcriptionally activates BBOX1-AS1 in NSCLC cells. a** Online JASPAR software was utilized to predict the possible binding sites of trascription factor KLF5 on BBOX1-AS1 promoter region. **b** GEPIA database displayed the expression profile of KLF5 in LUSC. **c** The correlation between BBOX1-AS1 and KLF5 expression was analyzed by bioinformatics tool GEPIA. **d** qRT-PCR assays were performed to determine the effect of KLF5 overexpression or knockdown on BBOX1-AS1 expression in A549 and SK-MES-1 cells. **e** ChIP assay was used to examine the binding of KLF5 with BBOX1-AS1 promoter at the predicted sites (P1 and P2). **f** The schematic diagram of luciferase reporter vectors containging wild type or mutant P2 sequence of BBOX1-AS1 promoter. **g** Dual luciferase reporter assay was conducted in A549 cells transfected with wt-P2 or mut-P2 reporter and vector or pcDNA-KLF5. **h** Dual luciferase reporter assay was performed in SK-MES-1 cells transfected with wt-P2 or mut-P2 reporter and si-NC or si-KLF5. ***P < 0.001

With the aim of exploring the potential mechanisms involved in BBOX1-AS1 up-regulation in NSCLC, JASPAR algorithm was used to predict the possible transcription factors that could bind to the promoter region of BBOX1-AS1. As shown in Fig. 2a, there were two putative binding sites for KLF5 in the BBOX1-AS1 promoter. GEPIA program displayed that KLF5 was highly expressed LUSC tumor tissues (Fig. 2b). Moreover, GEPIA website also found a positive correlation between BBOX1-AS1 and KLF5 in lung cancer tissues (Fig. 2c). Next, qRT-PCR was applied to detect the effect of KLF5 on BBOX1-AS1 expression in NSCLC cells. The results showed that BBOX1-AS1 expression was inhibited upon KLF5 knockdown, but was promoted due to KLF5 up-regulation in both A549 and SK-MES-1 cells (Fig. 2d). For exactly confirming the binding between KLF5 and BBOX1-AS1 promoter, ChIP assay followed by luciferase reporter experiments were carried out. ChIP assay results revealed that there was an occupancy for KLF5 at the P2 site (− 57 ~ − 48) on BBOX1-AS1 promoter (Fig. 2e). Afterwards, luciferase reporter vectors were constructed by inserting the wild type or mutant P2 sequence of BBOX1-AS1 promoter into pmirGLO, named as wt-P2 and mut-P2 (Fig. 2f). The luciferase reporter assay results manifested that overexpression of KLF5 in A549 cells significantly enhanced the luciferase activity of wt-P2 rather than mut-P2 (Fig. 2g). On the contrary, the luciferase activity of wt-P2 but not that of mut-P2 was decreased in SK-MES-1 cells with KLF5 knockdown (Fig. 2h). These data suggested that KLF5 contributed to the transcription activation of BBOX1-AS1.

### BBOX1-AS1 promotes cell malignant phenotypes in NSCLC

**Fig. 3:**
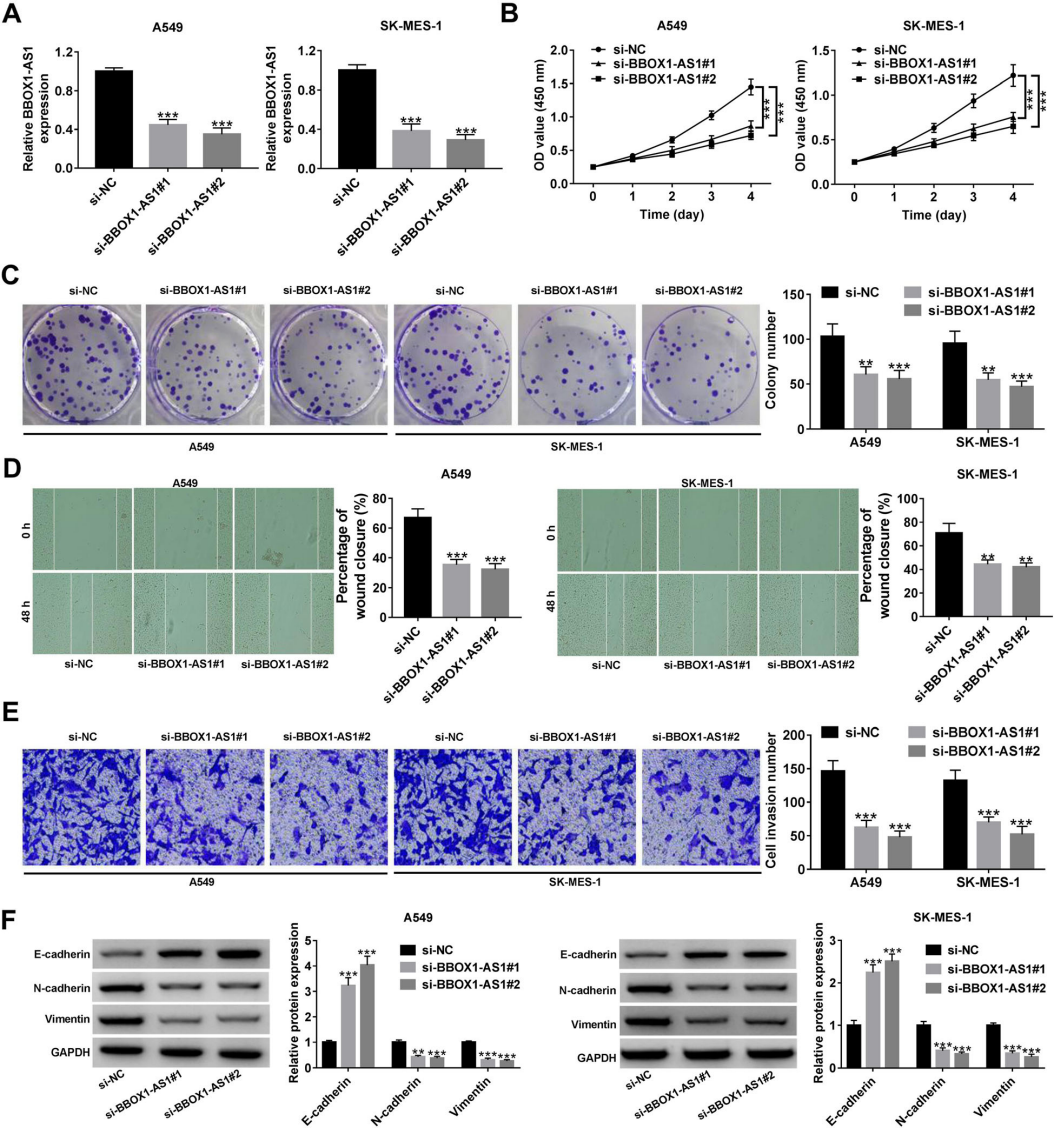
**BBOX1-AS1 induces cell proliferation, migration, invasion and EMT in NSCLC. a** After transfection with si-NC or siRNAs aganist BBOX1-AS1 (si-BBOX1-AS1#1 or si-BBOX1-AS1#2), NSCLC cells were subjected to qRT-PCR for detecting BBOX1-AS1 expression level. **b** and **c** CCK-8 and colony formation assays were carried out to assess cell proliferation potential upon BBOX1-AS1 knockdown. **d** Wound healing assays were used to evaluate the impact of BBOX1-AS1 depletion on cell migration. **e** Transwell assays were applied to detect cell invasion ability in the present of siRNAs targeting BBOX1-AS1. **f** Western blot assays were utilized to the measure the expression of EMT markers (E-cadherin, N-cadherin and vimentin) in NSCLC cells with low BBOX1-AS1 expression. **P < 0.01, ***P < 0.001

For the purpose of clarifying the biological functions of BBOX1-AS1 in NSCLC, two siRNAs specifically against BBOX1-AS1 (si-BBOX1-AS1#1 and si-BBOX1-AS1#2) were transfected into A549 and SK-MES-1 cells. As demonstrated by qRT-PCR, BBOX1-AS1 was effectively suppressed by these two siRNAs (Fig. 3a). Then, CCK-8 and colony forming experiments were performed to examine the influence of BBOX1-AS1 on cell proliferation. As presented in Fig. 3b and c, depletion of BBOX1-AS1 led to a significant repression of cell viability and colony forming ability. Subsequently, wound healing and transwell invasion assays were applied to evaluate the impact of BBOX1-AS1 on cell metastatic potential. Wound healing assays showed a decreased wound closure ability in A549 and SK-MES-1 cells with low BBOX1-AS1 expression (Fig. 3d). Transwell invasion assays revealed that the number of invasive cells was reduced due to BBOX1-AS1 knockdown (Fig. 3e). Meanwhile, western blot assays were used to determine the effect of BBOX1-AS1 on EMT. As a result, we observed an increase of E-cadherin expression, while a decline of N-cadherin and Vimentin expression in response to BBOX1-AS1 down-regulation (Fig. 3f). The aforementioned results indicated that BBOX1-AS1 facilitated cell proliferation, migration, invasion and EMT in NSCLC.

### BBOX1-AS1 serves AS a sponge for miR-27a-5p in NSCLC cells

**Fig. 4:**
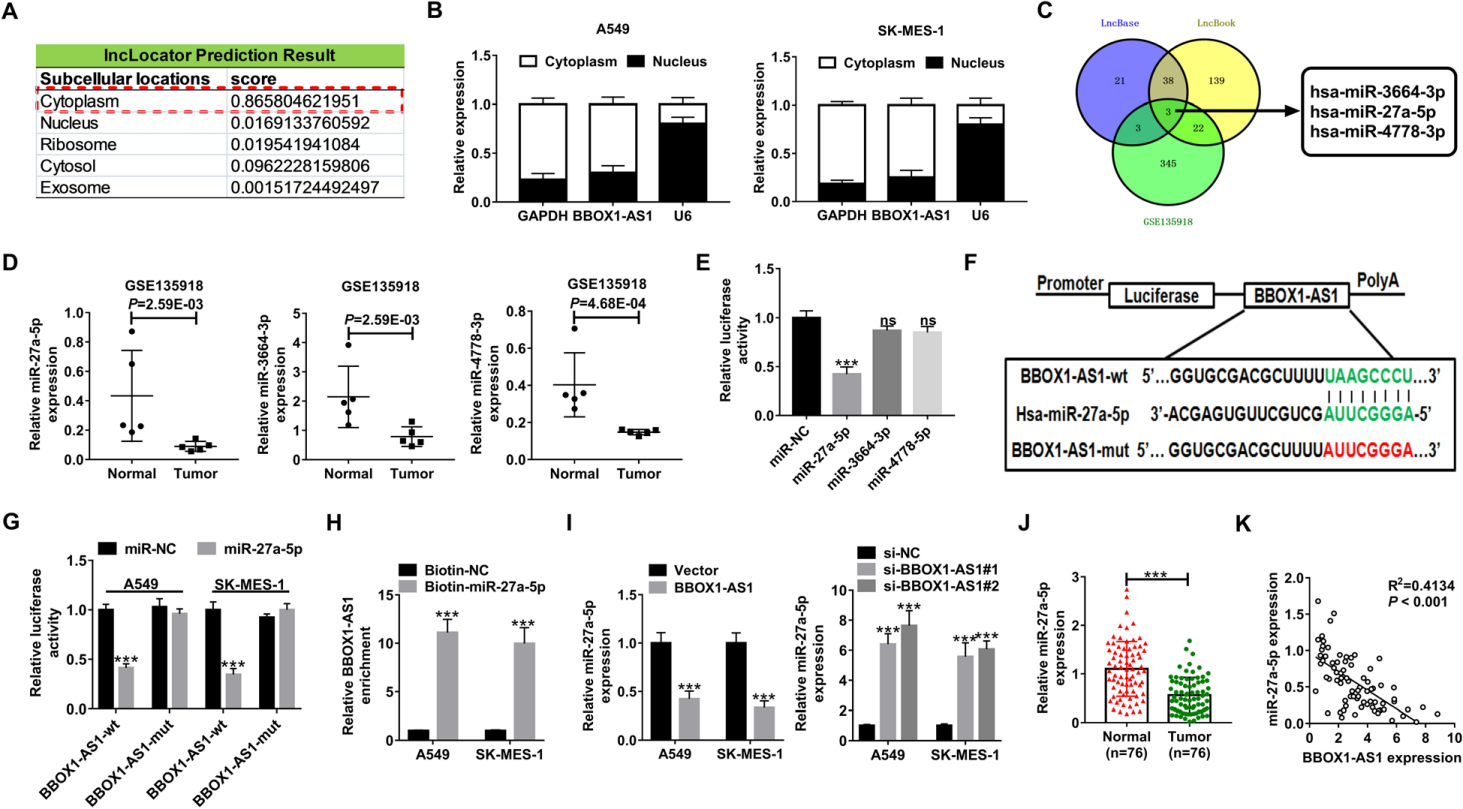
**BBOX1-AS1 interacts with miR-27a-5p to suppress its expression. a** lncLocator was used to predict the subcellular localization of BBOX1-AS1. **b** Subcellular fractionation assays were used to confirm the cytoplasm and nucleus distribution of BBOX1-AS1 in NSCLC cells. **c** Among the down-regulated miRNAs in NSCLC from GSE135918, three are found to be able to potentially interact with BBOX1-AS1 according to DIANA-LncBase and LncBook. **d** GSE135918 showed the expression of miR-27a-5p, miR-3664-3p and miR-4778-3p in 5 tumor tissues and matched normal tissues from NSCLC patients. **e** Dual-luciferase reporter assays were performed to detemine the influence of miR-27a-5p, miR-3664-3p and miR-4778-3p on the luciferase activity of BBOX1-AS1-wt reporter in HEK293T cells. **f** Schematic representation of wt- and mut-BBOX1-AS1 binding sequences on miR-27a-5p. Red fonts represented the mutant bases. **g** Dual-luciferase reporter assays were conducted in A549 and SK-MES-1 cells after transfection with BBOX1-AS1-wt or BBOX1-AS1-mut reporter and miR-NC or miR-27a-5p. **h** RNA pull-down assays were applied to examine the binding between miR-27a-5p and BBOX1-AS1 in NSCLC cells by using Biotin-NC or Biotin-miR-27a-5p. **i** The expression of miR-27a-5p was detected by qRT-PCR assays in NSCLC cells with BBOX1-AS1 overexpression or depletion. **j** qRT-PCR analysis of miR-27a-5p expression in NSCLC tumor tissues and corresponding non-cancerous. **k** Correlation analysis between miR-27a-5p and BBOX1-AS1 in NSCLC tumor specimens. ***P < 0.001

Next, we further made an attempt to uncover the mechanisms by which BBOX1-AS1 exerts oncogenic property in NSCLC. First of all, lncLocator online software was applied to predict the subcellular distribution of BBOX1-AS1. As delineated in Fig. 4a, BBOX1-AS1 was predominantly located in the cytoplasm. Subcellular fractionation experiments confirmed that there was much more BBOX1-AS1 in the cytoplasm of NSCLC cells than that in the nucleus (Fig. 4b), indicating the cytoplasmic localization of BBOX1-AS1 in NSCLC. One action mechanisms of cytoplasmic lncRNAs is that they are able to act as ceRNAs to sequester miRNAs and thus alleviate their suppression on target mRNAs [24]. Here, we speculated whether the carcinogenicity of BBOX1-AS1 in NSCLC was dependent on this “lncRNA-miRNA” interaction. By searching DIANA-LncBase v2.0 and LncBook programs, 41 miRNAs that contain the complementary sequences of BBOX1-AS1 were obtained. According to the data from gene chip GSE135918, a total of 373 down-regulated miRNAs were identified in lung cancer. Then, the candidate miRNAs obtained from these two methods were intersected. As shown in Fig. 4c, only three miRNAs (hsa-miR-3664-3p, hsa-miR-27a-5p and hsa-miR-4778-3p) were identified. GSE135918 showed a significant down-regulation of hsa-miR-3664-3p, hsa-miR-27a-5p and hsa-miR-4778-3p in lung cancer tissues compared with corresponding non-tumor tissues (Fig. 4d). By performing the luciferase reporter assays, miR-27a-5p suppressed the luciferase activity of wild-type BBOX1-AS1 reporter in HEK293T cells rather than miR-3664-3p or miR-4778-3p. Hence, we focus our attention on miR-27a-5p for further assays. The wild-type BBOX1-AS1 sequences harboring the miR-27a-5p binding sites as well as its mutant version in which the binding sequences were replaced were subcloned to luciferase reporter vector (Fig. 4f). Dual-luciferase reporter assays were conducted in A549 and SK-MES-1 cells co-transfected with miR-NC or miR-27a-5p and BBOX1-AS1-wt or BBOX1-AS1-mut reporter. The results showed that the luciferase activity of BBOX1-AS1-wt reporter but not that of BBOX1-AS1-mut was inhibited by miR-27a-5p overexpression in NSCLC cells (Fig. 4g). RNA pull-down assay disclosed that much more BBOX1-AS1 was captured by Biotin-miR-27a-5p rather than by Biotin-NC (Fig. 4h). Moreover, miR-27a-5p expression was decreased in NSCLC cells with BBOX1-AS1 up-regulation, while BBOX1-AS1 depletion was responsible for the increase of miR-27a-5p expression in both A549 and SK-MES-1 cells (Fig. 4i). Furthermore, qRT-PCR assays found that miR-27a-5p was down-regulated in NSCLC tumor tissues and inversely associated with BBOX1-AS1 expression (Fig. 4j and k). The starBase pan-cancer analysis platform described the consistent result (Additional File 1: Fig. S1B and C). According to OncoLnc (https://www.oncolnc.org/) that provides online survival analysis for the TCGA patients (490 LUAD cases and 466 LUSC cases), we found that there was no significant correlation between miR-27a-5p expression and patient survival in both LUAD and LUSC (Additional file 1: Fig. S1D). Consistently, no significant difference was observed for patient survival in our NSCLC cohort with high or low miR-27a-5p expression (Additional file 1: Fig. S1E). Thus, miR-27a-5p expression was not correlated with patient prognosis in NSCLC. To sum up, BBOX1-AS1 was able to interact with miR-27a-5p to inhibit its expression in NSCLC cells.

### BBOX1-AS1 accelerates NSCLC progression in vitro via sponging miR-27a-5p

**Fig. 5:**
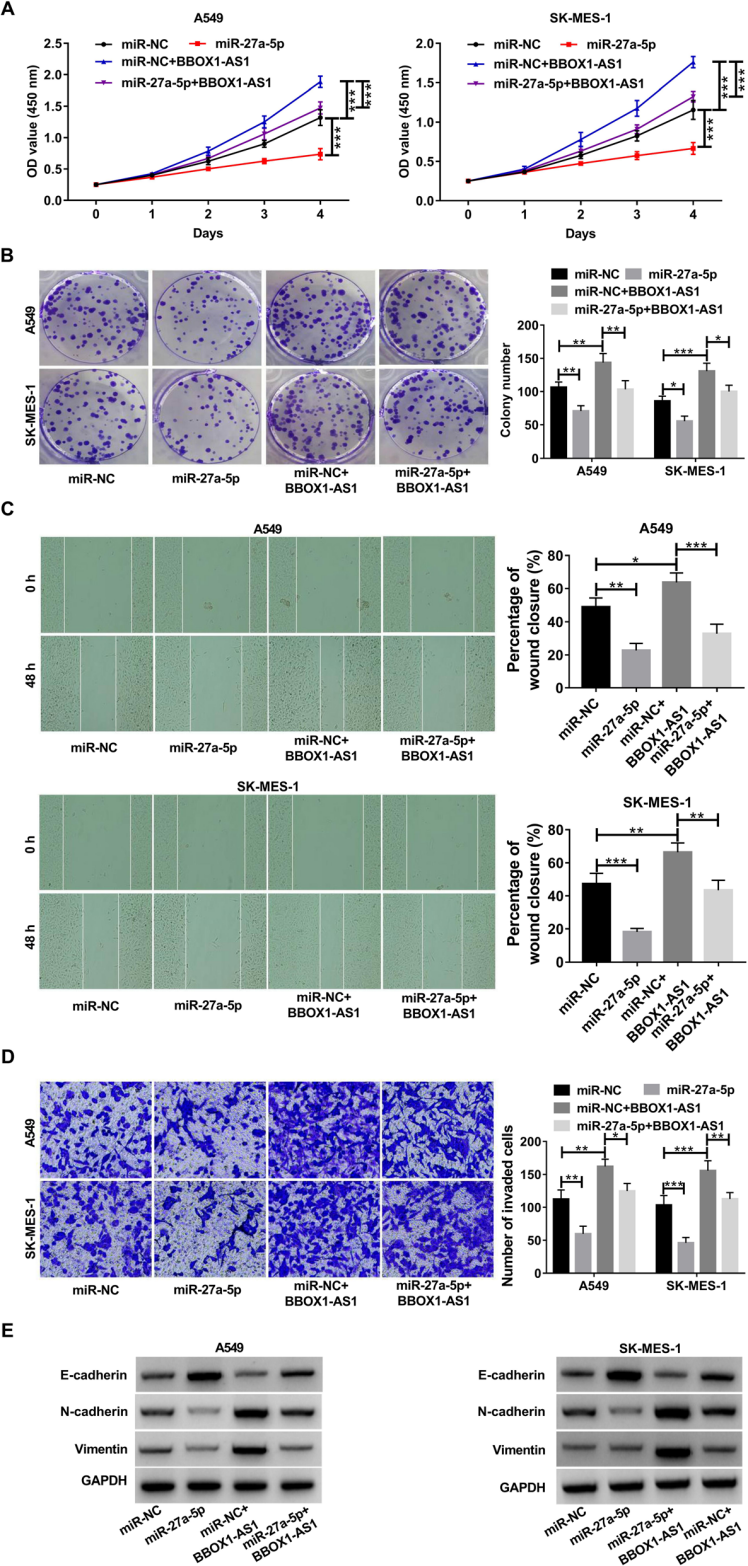
BBOX1-AS1 promotes NSCLC cell malignant phenotypes via sponging **miR-27a-5p. a**-**e** A549 and SK-MES-1 cells were transfected with miR-NC, miR-27a-5p, miR-NC + BBOX1-AS1 or miR-27a-5p + BBOX1-AS1, followed by CCK-8 assay of cell viability **a**, colony formation assay **b**, wound healing assay of cell migration **c**, transwell assay of cell invasion **d**, western blot assay of EMT-related markers **e**. *P < 0.05, **P < 0.01, ***P < 0.001.

Then, we further unearthed the role of miR-27a-5p in NSCLC, and whether miR-27a-5p was implicated in the regulation of BBOX1-AS1 in cell malignant behaviors. As a result, enforced expression of miR-27a-5p led to a significant repression of cell growth and colony forming capability (Fig. 5a and b). Moreover, cell migration and invasion potential was effectively attenuated in response to miR-27a-5p overexpression (Fig. 5c and d). Similarly, increased E-cadherin expression as well as decreased N-cadherin and Vimentin expression were found in NSCLC cells with miR-27a-5p up-regulation (Fig. 5e), suggesting the inhibitory effect of miR-27a-5p on EMT. As noted above, miR-27a-5p was a tumor-suppressive factor in NSCLC. Importantly, BBOX1-AS1-induced cell proliferation was greatly abrogated due to the overexpression of miR-27a-5p (Fig. 5a and b). Consistently, exogenous expression of miR-27a-5p also eliminated the promotive effects of BBOX1-AS1 on cell migration, invasion and EMT (Fig. 5c-e). These data supported that BBOX1-AS1 exerted tumor-promoting activity in NSCLC through modulating miR-27a-5p.

### BBOX1-AS1 up-regulates MELK expression via serving AS a sponge for miR-27a-5p

**Fig. 6:**
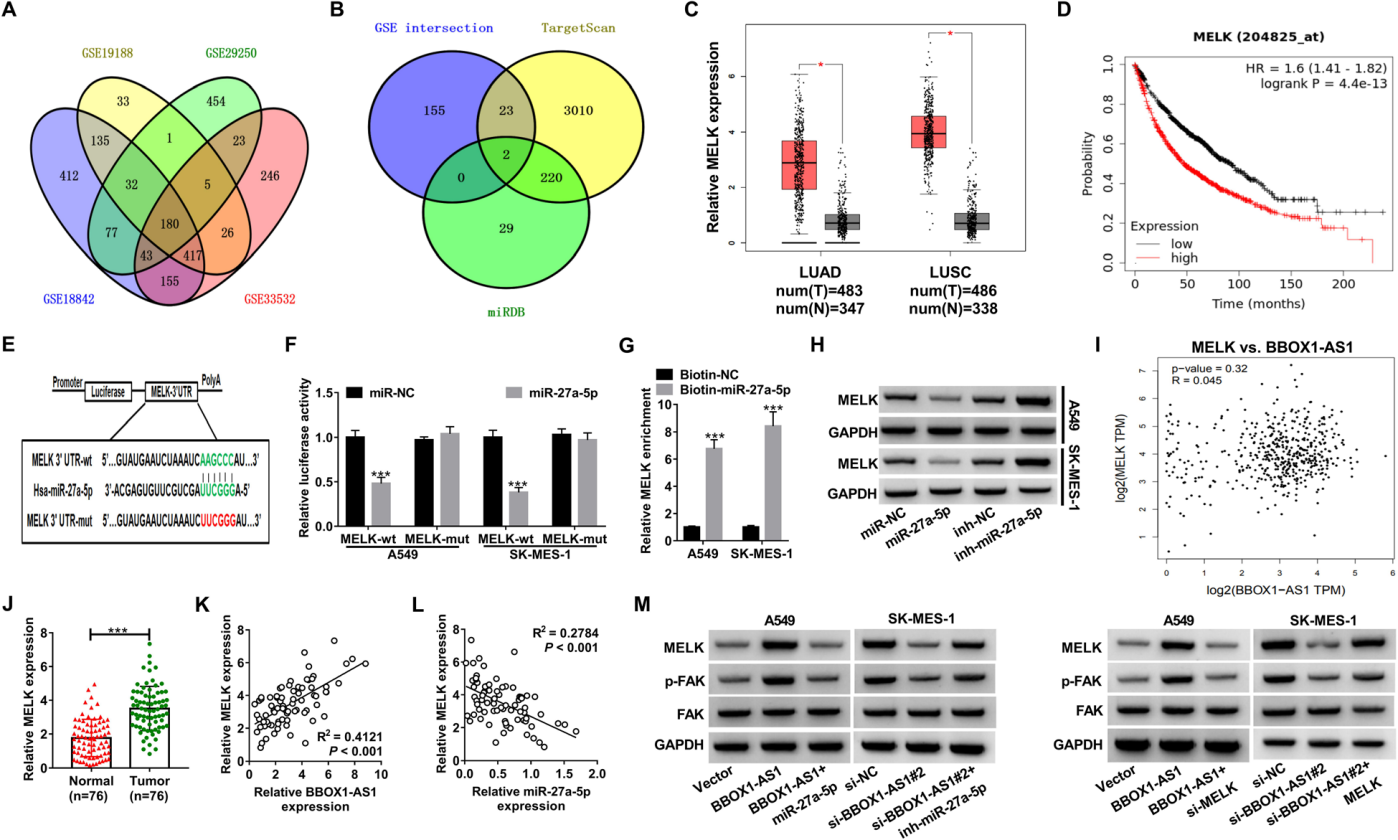
**BBOX1-AS1 regulates MELK expression through sponging miR-27a-5p in NSCLC cells. a** Venn diagram exhibited 180 up-regulated genes in NSCLC according to 4 different GEO datasets (GSE19188, GSE18842, GSE29250, GSE33532) following the criteria of log2 fold change ≥1. **b** Intersection of these 180 up-regulated genes and miR-27a-5p targets predicted by TargetScan and miRDB. **c** GEPIA was utilized to evaluate the expression of MELK in LUAD and LUSC. **d** Kaplan-Meier plotter database was used to analyze the prognostic significance of MELK in NSCLC patients. **e** Schematic diagram of the binding sites between miR-27a-5p and MELK-3’UTR. **f** Luciferase activity assays in A549 and SK-MES-1 cells transfected with miR-NC or miR-27a-5p and MELK-wt or MELK-mut reporter. **g** RNA pull-down assays were utilized to determine the enrichment of MELK mRNA in NSCLC cells after being precipitated by Biotin-miR-27a-5p. **h** Western blot assays were performed to determine the effect of miR-27a-5p mimic or inhibitor on MELK protein level in NSCLC cells. **i** Bioinformatics tool GEPIA showed a positive correlation between MELK and BBOX1-AS1 expression in lung cancer tissues. **j** qRT-PCR analysis of MELK expression in 76 NSCLC tumor tissues and adjacent normal samples. **k** Expression correlation between MELK and BBOX1-AS1 or miR-27a-5p in NSCLC tumor tissues. **l** The protein levels of MELK, p-FAK and FAK were determined by western blot assays in A549 cells transfected with Vector, BBOX1-AS1 or BBOX1-AS1 + miR-27a-5p and SK-MES-1 cells transfected with si-NC, si-BBOX1-AS1 or si-BBOX1-AS1 + inh-miR-27a-5p. ***P < 0.001

In order to make an investigation into the possible mechanism behind the inhibitory influence of miR-27a-5p on NSCLC, we collected the expression profile from 4 lung cancer microarrays (GSE19188, GSE18842, GSE29250 and GSE33532) to identify the differentially expressed genes in lung cancer. As depicted by Venn diagram in Fig. 6a, a total of 180 up-regulated genes were discovered in all datasets. By intersecting the above 180 up-regulated genes with the predicted targets of miR-27a-5p from TargetScan and miRDB, only two genes (MELK and PPAT) were screened out (Fig. 6b). According to a previous report, MELK was up-regulated in NSCLC and could potentially be used as a poor prognostic factor and therapeutic target for NSCLC [25]. Hence, we determined MELK as a follow-up research target. TCGA data from GEPIA and starBase Pan-Cancer Analysis Platform both disclosed higher expression of MELK in LUAD and LUSC tissues (Fig. 6c and Additional file 1: Fig. S1F). Moreover, patients with high MELK expression showed a significantly decreased overall survival compared to that with low MELK expression in lung cancer by Kaplan−Meier Plotter (Fig. 6d). The wild-type or mutant binding sites of MELK-3’UTR on miR-27a-5p were delineated in Fig. 6e. To validate the actual binding between miR-27a-5p and MELK-3’UTR, luciferase reporter and RNA pull-down experiments were performed. The results revealed that the luciferase activity of MELK-wt reporter was greatly suppressed due to miR-27a-5p overexpression, while no change was observed for the luciferase activity of MELK-mut reporter between miR-NC and miR-27a-5p (Fig. 6f). In contrast with Biotin-NC group, MELK was significantly enriched in RNA complex pulled down by Biotin-miR-27a-5p (Fig. 6g). Western blot assays manifested that overexpression of miR-27a-5p inhibited MELK protein level, while miR-27a-5p knockdown led to an increase of MELK protein expression in A549 and SK-MES-1 cells (Fig. 6h). It is thus apparent that MELK was a downstream target of miR-27a-5p in NSCLC cells.

Given that the ceRNA action mechanisms for lncRNAs, we further explored whether BBOX1-AS1 was able to affect MELK expression by sponging miR-27a-5p. Based on the TCGA data from GEPIA, there was a positive correlation between MELK and BBOX1-AS1 expression in LUSC tissues (Fig. 6i). qRT-PCR verified the higher MELK expression in NSCLC tumor samples than that in proximal normal tissues (Fig. 6j). Additionally, MELK level was positively correlated to BBOX1-AS1 expression, while was negatively associated with miR-27a-5p expression (Fig. 6k). What’s more, MELK protein level was increased in response to BBOX1-AS1 up-regulation in A549 cells, but this effect was impaired by miR-27a-5p overexpression or MELK knockdown (Fig. 6l and m). On the contrary, MELK protein level was reduced in SK-MES-1 cells due to BBOX1-AS1 silencing, while co-transfection with miR-27a-5p inhibitor or MELK-overexpressing plasmid reversed this influence (Fig. 6l and m). Focal adhesion kinase (FAK), a protein tyrosine kinase activated and/or overexpressed in advanced cancers, is able to drive tumor growth and metastasis [26]. Here, we found that BBOX1-AS1 overexpression promoted the phosphorylation levels of FAK in A549 cells, but miR-27a-5p up-regulation or MELK depletion abated this impact (Fig. 6l and m). In SK-MES-1 cells, knockdown of BBOX1-AS1 inhibited the phosphorylation levels of FAK, while this effect was abolished by miR-27a-5p down-regulation or MELK overexpression to a great extent (Fig. 6l and m). According to these data, it was concluded that BBOX1-AS1 served as a miR-27a-5p sponge to up-regulate MELK expression and activate FAK signaling.

### BBOX1-AS1 exerts oncogenic role in NSCLC via regulating MELK

**Fig. 7:**
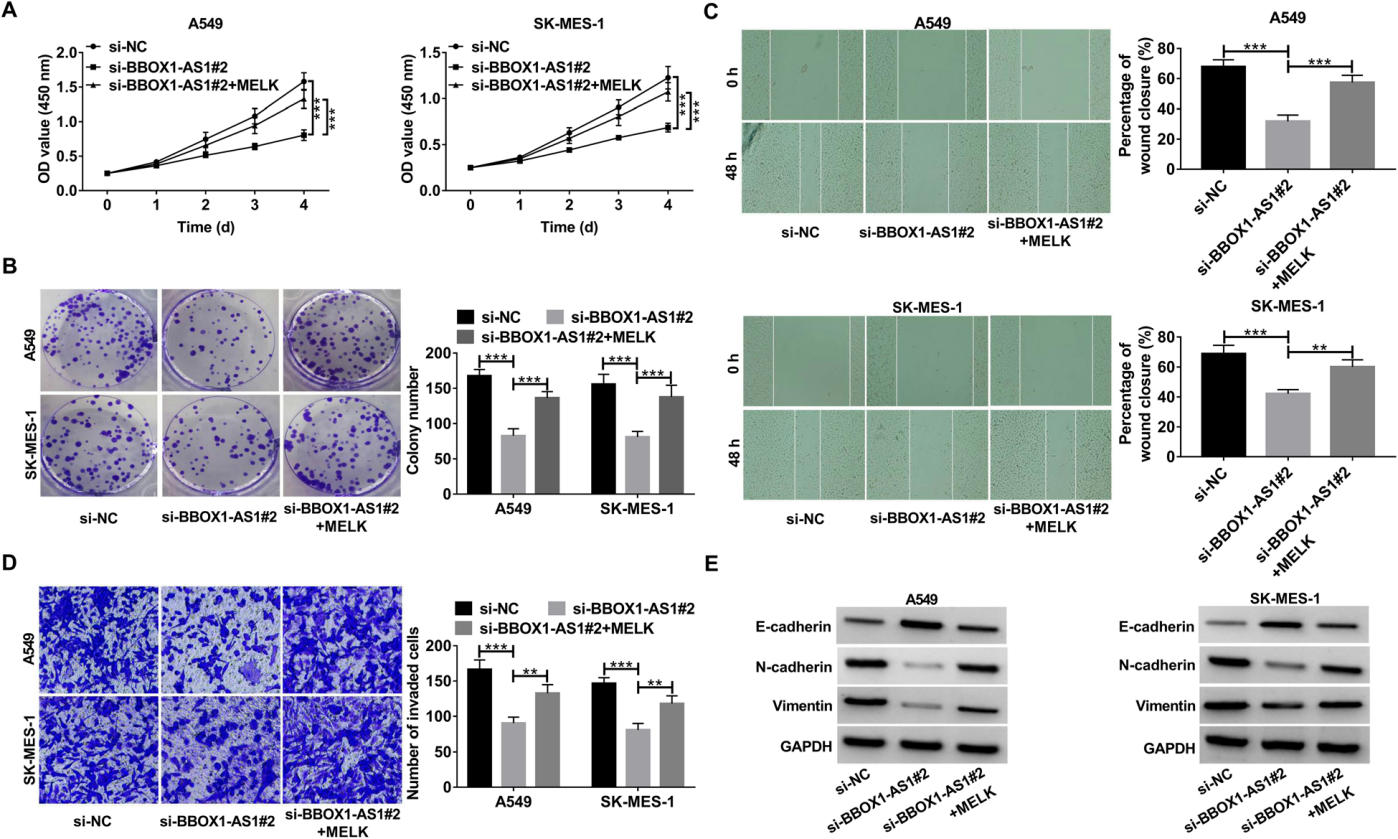
**BBOX1-AS1 facilitates the malignant progression of NSCLC by regulating MELK expression. a-e** A549 and SK-MES-1 cells were transfected with si-NC, si-BBOX1-AS1#2 or si-BBOX1-AS1#2 + MELK. **a** CCK-8 assays were used to determine cell viability. **b** Colony formation assays in transfected cells. **c** Wound healing assays were performed to measure cell migration capability. **d** Transwell assays were utilized to detect cell invasion ability. **e** Western blot assays were conducted to exaine the protein levels of E-cadherin, N-cadherin and vimentin. **P < 0.01, ***P < 0.001

In line with a previous document [27], knockdown of MELK was found to exert an inhibitory effect on NSCLC cell proliferation, migration and invasion (Additional file 1: Fig. S2A-D). In an effort to figure out whether BBOX1-AS1-mediated oncogenic activity in NSCLC was dependent on MELK, si-BBOX1-AS1#2 was transfected into A549 and SK-MES-1 cells alone or together with MELK-overexpressing vector. It turned out that the proliferation inhibition triggered by BBOX1-AS depletion was restored upon MELK up-regulation (Fig. 7a and b). Likewise, si-BBOX1-AS1-induced suppression of cell migration, invasion and EMT were counteracted in response to MELK overexpression (Fig. 7c-e).

**Fig. 8:**
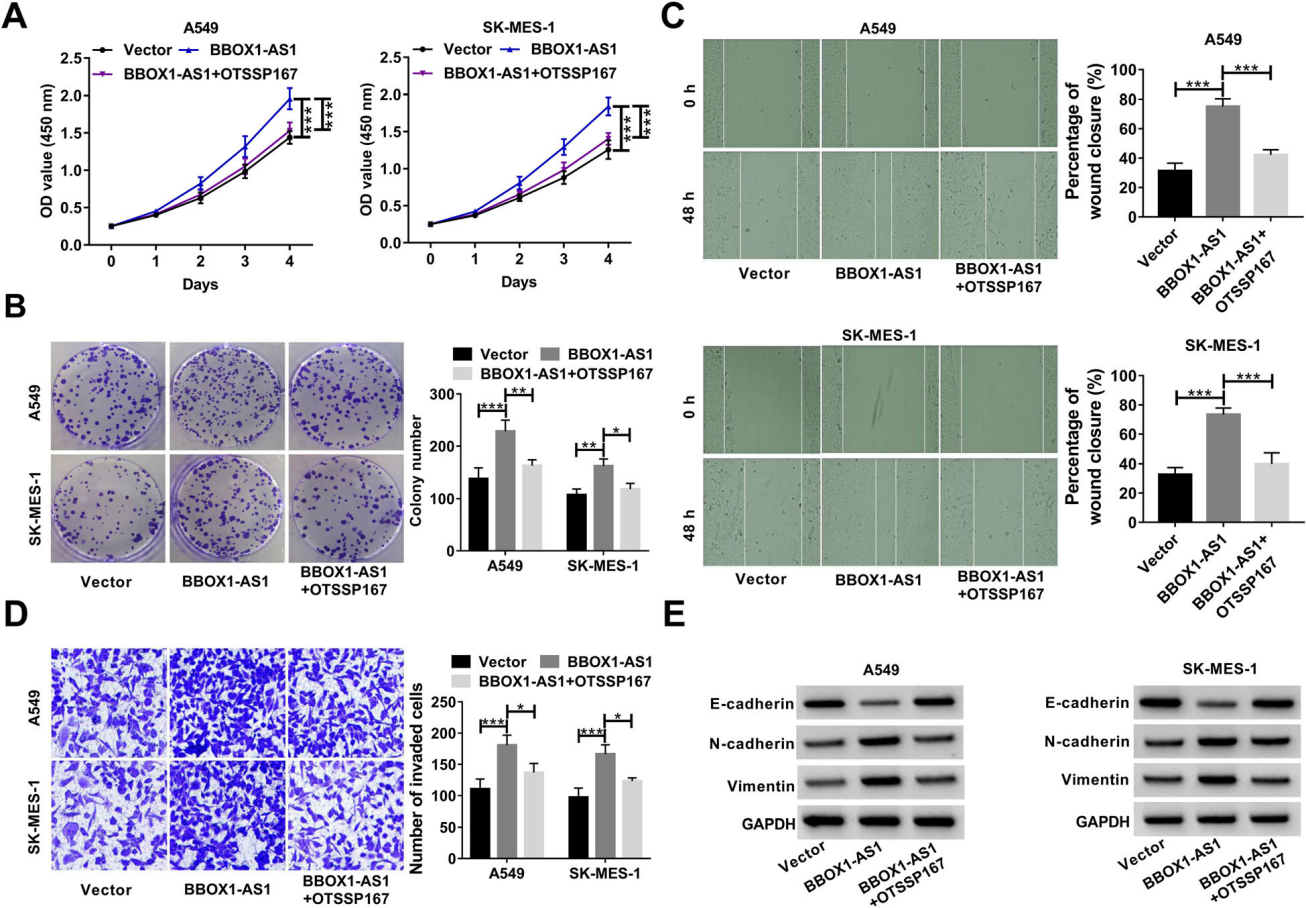
**MELK inhibitor (OTSSP167) attenuates BBOX1-AS1-mediated carcinogenicity in NSCLC cells. a-e** OTSSP167 (50 nM) was used to treat A549 and SK-MES-1 cells prior to transfection with pcDNA-MELK. **a** Cell viability was determined by CCK-8 assays. **b** Colony formation assays were used to assess cell proliferation ability. **c** and **d** Wound healing and transwell assays were applied to evaluate cell migration and invasion. **e** Western blot assays were performed to detect EMT-related protein expression. *P < 0.05, **P < 0.01, ***P < 0.001

On the other hand, a specific MELK inhibitor OTSSP167 (50 nM) was used to treat A549 and SK-MES-1 cells prior to transfection with BBOX1-AS1-overexpressing plasmid. The result showed that BBOX1-AS1-induced increase of cell viability and colony forming potential was reversed by treatment with OTSSP167 (Fig. 8a and b). Moreover, BBOX1-AS1-mediated promotion of cell migration, invasion and EMT was attenuated upon OTSSP167 treatment (Fig. 8c-e). Collectively, the carcinogenicity of BBOX1-AS1 was at least, in part, able to be explained by the increase of MELK expression.

### BBOX1-AS1 promoted NSCLC tumor growth in vivo

**Fig. 9:**
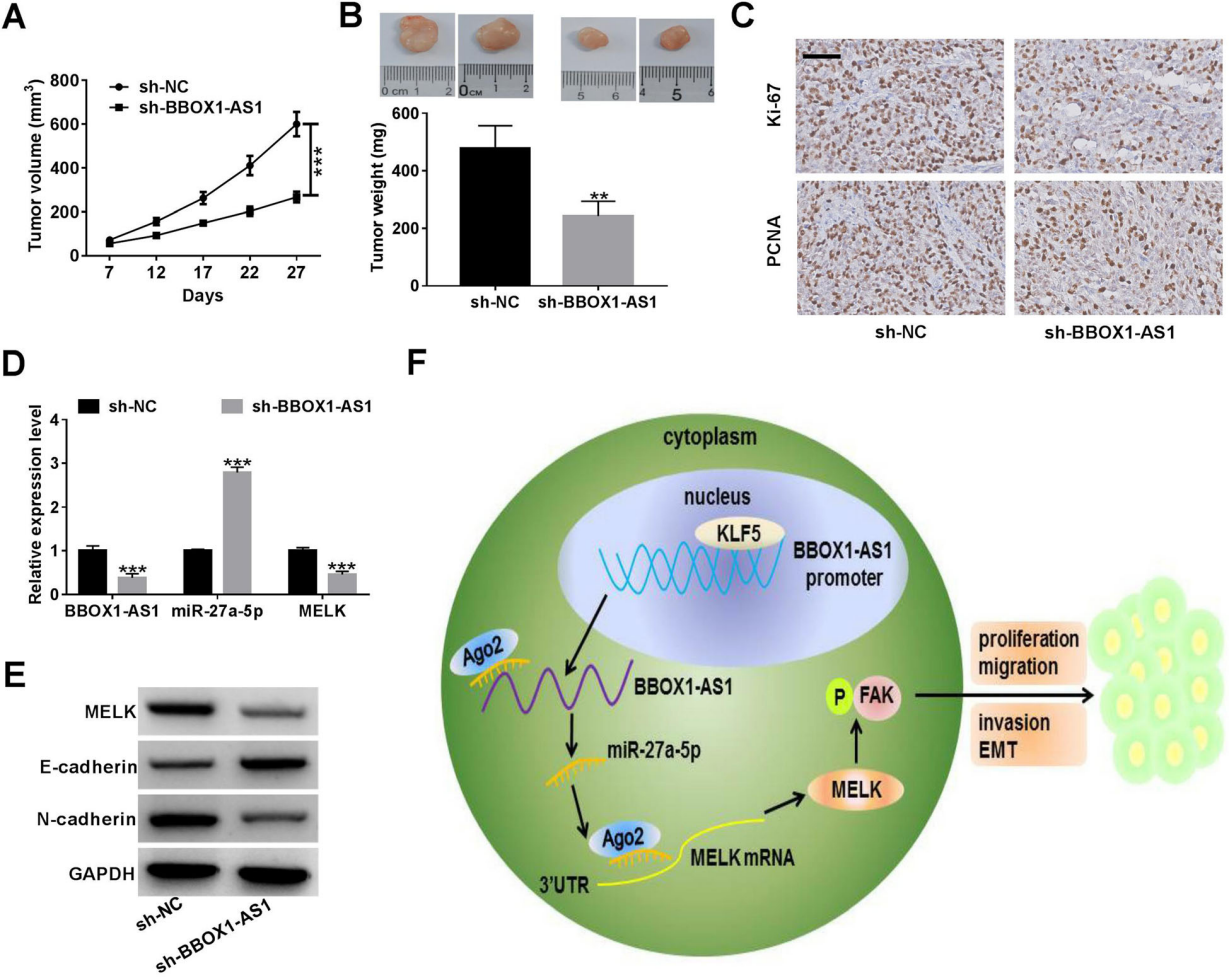
**Knockdown of BBOX1-AS1 suppresses NSCLC tumor growth in vivo.** A549 cells with lentivirus vectors carrying sh-BBOX1-AS1 or sh-NC were subcutaneously injected into the left armpit of mice. **a** Tumor volumes were monitored every 5 days. **b** Tumor weights were measured at the end of the experiments. **c** IHC staining were performed to determine the protein expression of proliferation index Ki-67 and PCNA in xenograft tumors. Scale bar, 50 μm. **d** qRT-PCR assays of BBOX1-AS1, miR-27a-5p and MELK mRNA expression in excised tumors. **e** Western blot assay of E-cadherin and N-cadherin protein expression in xenograft tumors. **f** Proposed mechanistic scheme. KLF5-induced BBOX1-AS1 acts as a sponge for miR-27a-5p to up-regulate MELK and activate FAK signaling, thereby promoting NSCLC cell proliferation, migration, invasion and EMT. **P < 0.01, ***P < 0.001

To examine the influence of BBOX1-AS1 on NSCLC tumor growth in vivo, A549 cells carrying sh-BBOX1-AS1 or sh-NC were subcutaneously injected into nude mice. The results showed that silencing of BBOX1-AS1 significantly suppressed tumor growth when compared with sh-NC group (Fig. 9a). As expected, tumor weight was lower in sh-BBOX1-AS1 group than that in sh-NC group (Fig. 9b). IHC analysis revealed a decline of Ki-67 and PCNA expression in xenograft tumor tissues derived from sh-BBOX1-AS1-transfected cells (Fig. 9c). qRT-PCR assay manifested that knockdown of BBOX1-AS1 led to a decrease of BBOX1-AS1 and MELK expression, while an enhancement of miR-27a-5p expression in tumor tissues (Fig. 9d). Western blot assays also demonstrated compared to sh-NC group, MELK protein level was declined in BBOX1-AS1-depletion tumor tissues (Fig. 9e). Moreover, inhibition of BBOX1-AS1 led to an increase of E-cadherin protein level, but a decrease of N-cadherin protein expression in the excised tumors (Fig. 9e). Altogether, BBOX1-AS1 was responsible for NSCLC tumorigenesis in vivo.

## Discussion

With the development of systemic therapies in NSCLC, such as targeted therapy, immunotherapy and chemotherapy, the clinical outcome for advanced and metastatic patients has been improved [28, 29]. In recent years, lncRNAs have drawn a great deal of attention and interest as crucial regulator in multiple cellular processes associated with tumorigenesis and metastasis [30, 31]. In the present study, we analyzed the expression profile of lncRNAs in NSCLC through two microarray datasets (GSE18842 and GSE19188). Aberrantly up-regulated BBOX1-AS1 was selected as the object for function and mechanism investigation. As a result, BBOX1-AS1 was confirmed to be highly expressed in NSCLC tumor tissues and cells. Moreover, high BBOX1-AS1 expression was positively related to tumor size, TNM stage and lymph node metastasis. Additionally, high BBOX1-AS1 expression was observed to indicate an adverse prognosis for NSCLC patients. These data presented above implied the potential application of BBOX1-AS1 as a prognosis biomarker for NSCLC.

Emerging documents demonstrate that some transcription factors are able to induce the transcription and expression of lncRNAs, such as SP1 [32] and EGR1 [33]. With the assistance of online tool JASPAR, KLF5 was predicted as a candidate transcription factor. By using ChIP and luciferase reporter assays, we confirmed that KLF5 was responsible for BBOX1-AS1 up-regulation in NSCLC cells by binding to its promoter region. In line with our data, KLF5 was also discovered as a transcription factor to facilitate lncRNAs expression in other malignancies. For instance, KLF5-activated lncRNA NEAT1 accelerated gastric cancer progression via serving as a scaffold for BRG1 to down-regulate GADD45A expression [34]. KLF5-regulated lncRNA RP1 contributed to cell proliferation and metastasis in breast cancer through suppressing p27kip1 translation [35]. Overall, the abnormal increase of BBOX1-AS1 was at least partly attributable to KLF5 dysregulation.

Recently, the biological functions of BBOX1-AS1 in several human cancers have been demonstrated. Yang et al. revealed that BBOX1-AS1 was up-regulated in gastric cancer, and promoted cell growth via interaction with miR-3940-3p to facilitate BIRC5 expression [16]. Xu et al. uncovered that BBOX1-AS1 expression was elevated in cervical cancer, and BBOX1-AS1 could drive cell malignant phenotypes by up-regulating HOXC6 via miR-361-3p and HuR [17]. Yao et al. found that BBOX1-AS1 exerted a tumor-promotive effect in ovarian cancer through sequestering miR-361-3p to enhance PODXL expression [18]. Liu et al. disclosed that BBOX1-AS1 acted as a sponge for miR-361-3p to induce SH2B1 expression, thereby accelerating cell proliferation and metastasis in colorectal cancer [19]. In the present study, we found that depletion of BBOX1-AS1 inhibited NSCLC cell proliferation, migration, invasion and EMT in vitro. Moreover, knockdown of BBOX1-AS1 slowed xenograft growth in vivo. All these results supported BBOX1-AS1 as a tumor driver in NSCLC progression.

A hypothesis that cytoplasmic lncRNAs can compete for miRNAs via binding to their shared binding sites on protein-coding mRNAs has been widely accepted [36]. By bioinformatic prediction tool and subcellular fractionation assay, most of BBOX1-AS1 was confirmed to be located in the cytoplasm. According to web-based algorithms, luciferase reporter assays and RNA pull down experiments, miR-27a-5p was validated as a downstream target of BBOX1-AS1. MiR-27a is demonstrated to be implicated in oncogenesis, invasion, metastasis, EMT and chemoresistance in kinds of solid tumors, implying its potential as biomarker and therapeutic target in human cancers [37, 38]. A previous document confirmed the downregulation of miR-27a-5p in small cell lung cancer (SCLC) clinical specimens, and ectopic expression of miR-27a-5p suppressed cancer cell aggressiveness [39]. What’s more, a recent document revealed that miR-27a-5p was down-regulated in lung adenocarcinoma and exerted an tumor-suppressive activity via inhibiting cell colony forming ability [40]. Nonetheless, there is still an insufficient understanding of role miR-27a-5p’s role in NSCLC development. In this current study, we found that miR-27a-5p overexpression significantly suppressed proliferation, migration, invasion and EMT of both A549 and SK-MES-1 cells. More importantly, miR-27a-5p could partly countervail the promotive effects of BBOX1-AS1 on NSCLC cell malignant phenotypes. Therefore, it was inferred that BBOX1-AS1 could act as an endogenous sponge to repress the expression and function of miR-27a-5p.

MELK, a highly conserved serine/threonine kinase, has been reported to be highly expressed in various types of human malignancies and exert an oncogenic role through modulating cell proliferation, apoptosis, cancer stem cell phenotypes, EMT, metastasis and treatment resistance [41]. In accordance with previous literatures concerning MELK in NSCLC [25, 42], we found that MELK was up-regulated in NSCLC tumor samples compared with normal specimens by analyzing the lung carcinoma mRNA microarray profile from the GEO datasets. Through a series of bioinformatic prediction and experiment validation, MELK was identified as a direct target of miR-27a-5p. Functionally, silencing of MELK resulted in a significant repression of cell proliferation, migration and invasion in NSCLC. Consistently, Tang et al. recently uncovered MELK as a oncogenic kinase critical for metastasis, mitotic progression and programmed death of LUAD [27]. Mechanistically, BBOX1-AS1 served as a molecular sponge for miR-27a-5p to attenuate its suppressive effects on MELK expression. Furthermore, the tumor-suppressive activity owing to BBOX1-AS1 knockdown was partly reversed by MELK overexpression. Focal adhesion kinase (FAK), a cytoplasmic protein tyrosine kinase that is overexpressed and activated in several solid cancers, could drive tumor progression and metastasis through different pathways [43]. Here, we found that BBOX1-AS1 could activate FAK signaling by sponging miR-27a-5p. Thus, we concluded that BBOX1-AS exerted oncogenic effects in NSCLC at least in part by positively regulating MELK/FAK pathway via sponging miR-27a-5p. Liu et al. recently disclosed that MELK facilitated colorectal cancer progression by activating FAK/Src pathway [44]. MELK is reported to be involved in multiple cellular events in tumorigenesis and malignant progression of human cancers by phosphorylation and regulation of several signaling molecules [45], such as NF-*κ*B [46] and mTOR [47]. Thus, more detailed investigation on the downstream signaling pathways of MELK is required in NSCLC.

## Conclusion

Taken together, BBOX1-AS1 was highly expressed in NSCLC. Higher BBOX1-AS1 expression was correlated with worse clinical parameters and poorer patient outcomes. Moreover, BBOX1-AS1 up-regulation in NSCLC was induced by transcription factor KLF5. Functionally, knockdown of BBOX1-AS1 inhibited cell proliferation, migration, invasion and EMT in vitro and slowered tumor growth in vivo. Mechanistically, BBOX1-AS1 acted as a sponge for miR-27a-5p to release MELK expression and activating FAK signaling. Our findings clarified a KLF5/BBOX1-AS1/miR-27a-5p/MELK regulatory axis in NSCLC (Fig. 8f), offering a theoretical foundation for employing BBOX1-AS1 as a promising therapeutic target for NSCLC. Although lncRNA-based researches open up new avenues for therapeutic intervention of cancer patients, there is still a long way to go to bring these molecular targets to clinic use.

## Supplementary Information

**Additional file 1: Table S1.** Primer sequences used in qRT-PCR. **Figure S1.** (A) GEPIA website shows the expression of BBOX1-AS1 in LUSC. (B) starBase Pan-Cancer Analysis Platform reveals the expression of miR-27a-5p in LUSC. (C) starBase Pan-Cancer Analysis Platform was used to analyze the correlation between miR-27a-5p and BBOX1-AS1 in lung cancer tissues. (D) Kaplan-Meier survival plots using the TCGA LUAD and LUSC patients by expression value of miR-27a-5p. (E) Kaplan-Meier survival curve was used to determine the correlation between overall survival and miR-27a-5p expression in NSCLC. (F) GEPIA database displays the expression of MELK mRNA in LUSC and LUAD. **Figure S2.** MELK promotes cell proliferation, migration and invasion in NSCLC. (A-D) A549 and SK-MES-1 cells were transfected with si-NC or si-MELK, followed by CCK-8 assay of cell viability (A), colony forming assay (B), wound healing assay of cell migration (C) and transwell assay of cell invasion. **P < 0.01, ***P < 0.001.

## Data Availability

The datasets used and analysed during the current study are available from the corresponding author on reasonable request.

## Abbreviations

NSCLC: Non-small cell lung cancer
lncRNAs: Long non-coding RNAs
BBOX1-AS1: BBOX1 anti-sense RNA 1
MELK: Maternal embryonic leucine zipper kinase
LUAD: Lung adenocarcinoma
LUSC: Lung squamous cell carcinoma
EMT: Epithelial to mesenchymal transition
ceRNAs: Competing endogenous RNAs
siRNAs: Small interfering RNAs
qRT-PCR: Quantitative real-time PCR
ChIP: Chromatin immunoprecipitation
IHC: Immunohistochemistry
ANOVA: One-way analysis of variance
FAK: Focal adhesion kinase
