# Supplementary material for: KLF5-induced BBOX1-AS1 contributes to cell malignant phenotypes in non-small cell lung cancer via sponging miR-27a-5p to up-regulate MELK and activate FAK signaling pathway
Source: J Exp Clin Cancer Res. 2021 Apr 30;40:148. doi: 10.1186/s13046-021-01943-5 (PMC8086369; doi:10.1186/s13046-021-01943-5)
Supplement: Supplementary file 1 — Additional file 1: Table S1. Primer sequences used in qRT-PCR. Figure S1. (A) GEPIA website shows the expression of BBOX1-AS1 in LUSC. (B) starBase Pan-Cancer Analysis Platform reveals the expression of miR-27a-5p in LUSC. (C) starBase Pan-Cancer Analysis Platform was used to analyze the correlation between miR-27a-5p and BBOX1-AS1 in lung cancer tissues. (D) Kaplan-Meier survival plots using the TCGA LUAD and LUSC patients by expression value of miR-27a-5p. (E) Kaplan-Meier survival curve was used to determine the correlation between overall survival and miR-27a-5p expression in NSCLC. (F) GEPIA database displays the expression of MELK mRNA in LUSC and LUAD. Figure S2. MELK promotes cell proliferation, migration and invasion in NSCLC. (A-D) A549 and SK-MES-1 cells were transfected with si-NC or si-MELK, followed by CCK-8 assay of cell viability (A), colony forming assay (B), wound healing assay of cell migration (C) and transwell assay of cell invasion. **P < 0.01, ***P < 0.001. [file 13046_2021_1943_MOESM1_ESM.docx]

**Table S1 Primer sequences used in qRT-PCR**

| Genes | Primer sequences (5’-3’) |
| --- | --- |
| BBOX1-AS1 | F: CAGACTCCTGCTTTGCTCTT |
|  | R: GGAAGCATCTTCTCAGCTTCT |
| MELK | F: GCCTGCCATATCCTTACTGG |
|  | R: TGGCTGTCTCTAGCACATGG |
| miR-27a-5p | F: TGTATTTTAGTCGTGGCGATA |
|  | R: ATAACGACTCACGCCTATAATC |
| GAPDH | F: AGCCACATCGCTCAGACAC |
|  | R: GCCCAATACGACCAAATCC |
| U6 | F: ATTGGAACGATACAGAGAAGATT |
|  | R: GGAACGCTTCACGAATTTG |

**Figure S1**

**
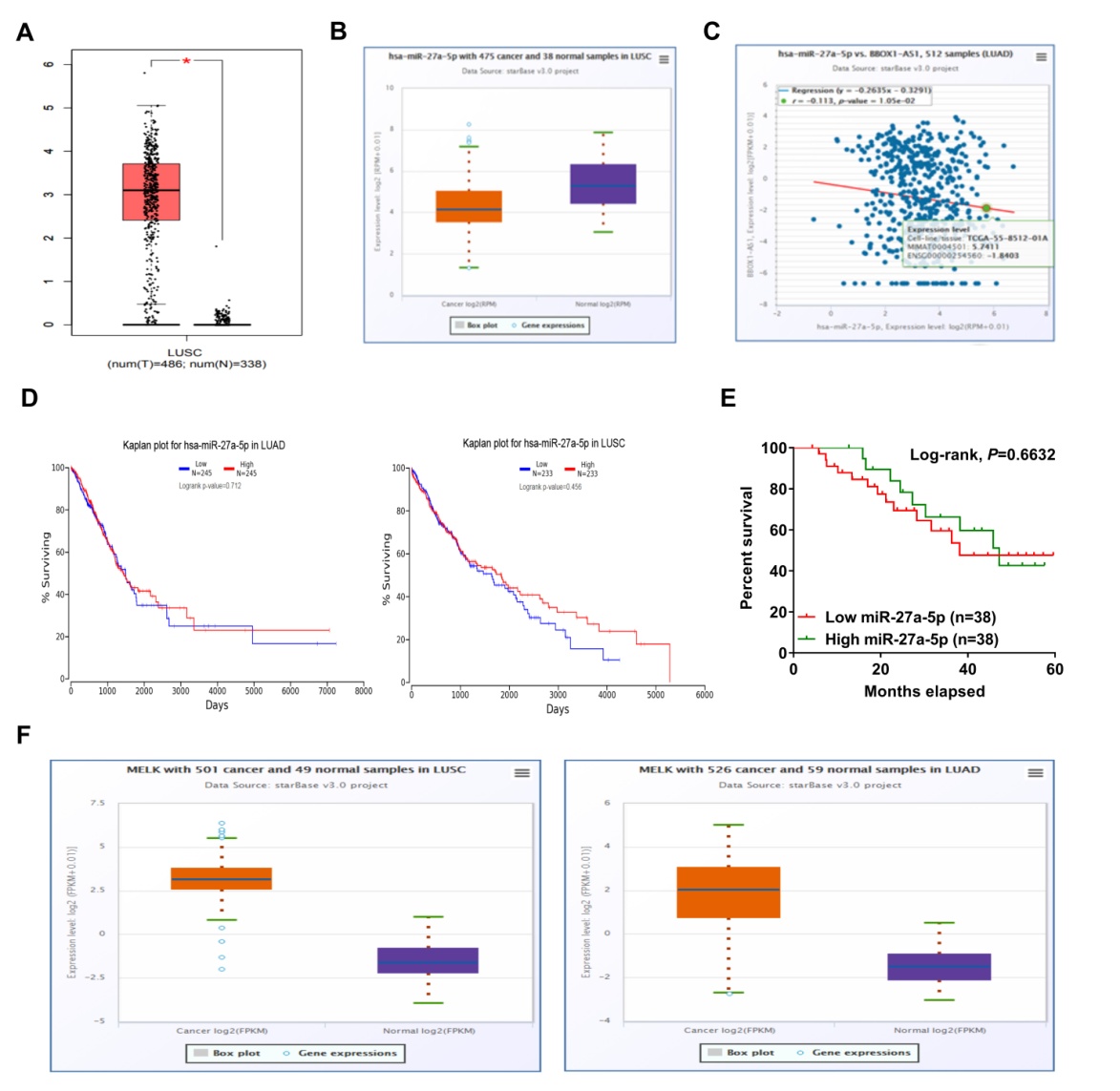
**

**Figure S1** (A) GEPIA website shows the expression of BBOX1-AS1 in LUSC. (B) starBase Pan-Cancer Analysis Platform reveals the expression of miR-27a-5p in LUSC. (C) starBase Pan-Cancer Analysis Platform was used to analyze the correlation between miR-27a-5p and BBOX1-AS1 in lung cancer tissues. (D) Kaplan-Meier survival plots using the TCGA LUAD and LUSC patients by expression value of miR-27a-5p. (E) Kaplan-Meier survival curve was used to determine the correlation between overall survival and miR-27a-5p expression in NSCLC. (F) GEPIA database displays the expression of MELK mRNA in LUSC and LUAD.

**Figure S2**


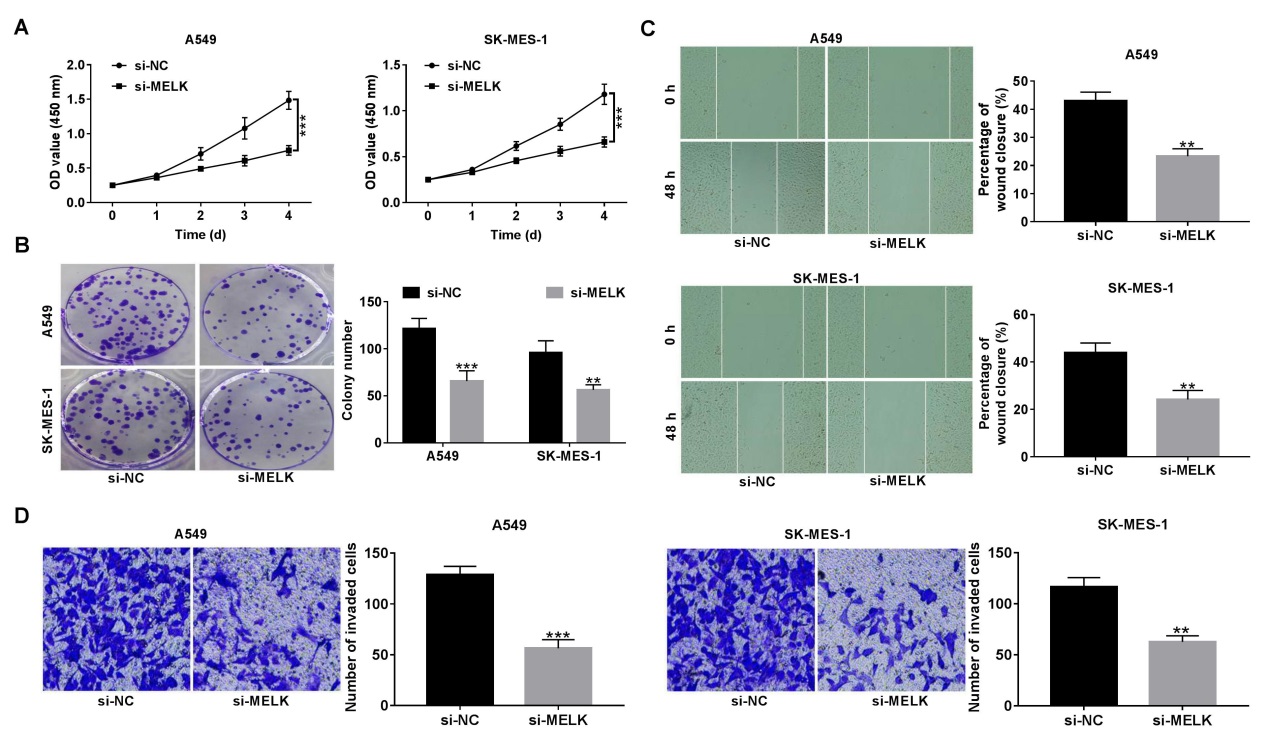


**Figure S2 MELK promotes cell proliferation, migration and invasion in NSCLC.** (A-D) A549 and SK-MES-1 cells were transfected with si-NC or si-MELK, followed by CCK-8 assay of cell viability (A), colony forming assay (B), wound healing assay of cell migration (C) and transwell assay of cell invasion. ***P* < 0.01, ****P* < 0.001.
